# Monthly Summary

**Published:** 1886-03

**Authors:** 


### Monthly Summary.

---

CAPSICUM BAGS.—*Dr. Ingersoll.*—The origin of this appliance was, I believe, with a young lady who had acquired the art of facial expression by gum-chewing. She knew that mustard was a good thing to relieve pains, and wanted to try the application of mustard to an aching tooth. She took a piece of chewing-gum, and made it like the bowl of a spoon, then, putting the mustard in it, she placed the gum back in her mouth, and pressed it down over the deceased part; now she had a mustard plaster applied to the tooth. From that I believe, originated the capsicum bag, one side of which is linen, the other oiled silk. They are filled with capsicum, or capsicum and ginger, and are applied without danger of vesicating. I tried these, and found that they moved about from place to place, and would not bring about the desired results. Then—I do not know where I got the idea, but it was from somebody wise enough to know what to do. I wish I could remember, to give the man credit for it, It is this: I take the extract of capsicum—I do not like the tincture, it is too alcoholic—the extract is more aqueous; I take the very heaviest bibulous paper, after having examined to see what size I want; I tear a piece off—I prefer to tear it; if you cut it with scissors, the edge will be stiff and hard; If it is torn off, it leaves a feathery edge, and the movement of the cheek will not so readily move it out of place. Take a piece of cotton and dip it in the extract of capsicum, and with it saturate one side of the bibulous paper; then turn it over, and cover the other side with thick sandarac varnish—as thick as cream. This apply to the tooth, the varnished side toward the cheek. I hold it there until the saliva has saturated it so to retain it. In that condition it will adhere quite closely to the gnm; the outside being covered with gum sandarac, it does not affect the mucous membrane of the cheek. This plan I have found to operate more surely than anything else, Capsicum is the most powerful of stimulents, as well as the most persistent; almost any other will lose its power in an hour, but this will contioue for hours as pungent and as active as ever. It is a most persistent irritant. It promotes circulation; and nature, in these cases, aided by this stimulant, is frequently able to carry off formed pus and effect a cure

---

TO MEND A BROKEN FILE.—Take a file as soon as it is broken, and wet the break with zinc dissolved in muriatic acid, and then tin over with the soldering iron. This must be done immediately, as soon as the file is broken, as the break begins to oxidize when exposed to the air, and in an hour or two will gather sufficient to make it impossible for the parts to adhere. Heat the file as warm as it will bear without disturbing its temper as soon as well tinned, and press the two pieces firmly together, squeezing out nearly all the solder, and hold in place till the file cools. This can be done with very little to trim off, and every portion of the break fitting accurately in place. Bring both pieces in line with each other, and, for a file, it is as strong in one place as in another. and is all that could be asked for under the very best of welding treatment.—*Popular Science News.*

---

GLYCERINE IN DRYNESS OF THE TONGUE.—The annoying dryness of the tongue and pharynx, so common in acute febrile diseases, according to Cotter, may be relieved by brushing pure and undiluted glycerine over the affected parts. The great thirst ceases, and the patient's condition is made more comfortable. It is probable that glycerine stimulates the secretion of the salivary glands, and thus acts as a solvent for inspissated material accumulating upon the buccal mucous membrane in cases of this kind.

---

PRESERVATION OF COCAINE SOLUTIONS.—Cocaine is well known to be liable, when in solution, to be altered by the growth of microscopic fungi, so as to produce severe local inflammatory symptoms. Dr. Squibb, of Brooklyn, after trying various agents to prevent this change, finds that salicylic acid answers best for the purpose. This is added to the alkaloidal salt in the proportion of one six-hundredth part, this quantity being sufficient to protect the solution indefinitely. Salicylic acid is soluble in about three hundred parts of water: and it is advantageous to keep the solution ready-made, and use half of water and half of solution of salicylic acid, for the purpose of dissolving the cocaine salt.

